# The Role of Free Will Belief, Gratitude, and Self‐Control in Improving Nurses’ Job Performance: A Cross‐Sectional Study of a Multiple Mediation Model

**DOI:** 10.1155/jonm/9002280

**Published:** 2025-12-08

**Authors:** Linli Zhuang, Xueling Suo, Song Wang, Martin Cerveny

**Affiliations:** ^1^ Department of Rheumatology and Immunology, West China Hospital, Sichuan University, Chengdu, China, scu.edu.cn; ^2^ West China School of Nursing, Sichuan University, Chengdu, China, scu.edu.cn; ^3^ Department of Radiology, West China Hospital, Sichuan University, Chengdu, China, scu.edu.cn

**Keywords:** belief in free will, Chinese nurses, gratitude, job performance, self-control

## Abstract

**Aims:**

The present study aimed to examine the relationships between belief in free will (BFW), gratitude, self‐control, and job performance among nurses, with a particular focus on the mediating roles of gratitude and self‐control.

**Background:**

Job performance is central to human resource management, particularly in nursing, where it has a significant impact on productivity, quality of care, and patient safety. Recent research has highlighted the importance of positive psychological beliefs (e.g., BFW) in an individual’s job performance. However, little is known about the underlying psychological mechanisms linking BFW to job performance.

**Design:**

A quantitative cross‐sectional survey design was used for this research.

**Methods:**

Conducted from January 2021 to May 2022 in two southwest cities of China, the study involved a convenience sample of 709 nurses from several hospitals. Participants completed an anonymous online questionnaire that included measures of BFW, gratitude, self‐control, and job performance. Data analysis was performed with IBM SPSS 26.0 and Hayes’ PROCESS macro, using multiple mediation analyses to examine the relationships and mediating effects between variables.

**Results:**

The study found significant positive correlations between BFW, gratitude, self‐control, and job performance. Multiple mediation analyses indicated that both gratitude and self‐control significantly mediated the relationship between BFW and job performance. The sequential mediation effect of gratitude and self‐control on the relationship between BFW and job performance was also statistically significant.

**Conclusions:**

BFW may have a positive effect on job performance among nurses, with gratitude and self‐control serving as independent and serial mediators. Enhancing these psychological factors may improve nurses’ job performance.

**Implications for Nursing Management:**

Understanding the impact of psychosocial factors such as BFW, gratitude, and self‐control on job performance can help nurse managers develop strategies to improve staff performance and the quality of patient care. By fostering a positive psychological environment, nurse managers can motivate nurses to higher levels of performance.

**Reporting Method:**

The STROBE criteria were used to report the survey results.

## 1. Introduction

Job performance, a key concept in human resource management, refers to the measurement of how well an individual carries out his/her tasks in the workplace [1]. It involves performing tasks effectively, demonstrating contextual behaviors, and using adaptive skills that contribute to a positive work environment. Improving the performance of health workers is essential to meeting the challenges of maintaining accessible and cost‐effective health services [2]. In particular, nurses’ job performance is an important predictor of productivity, quality of care, and patient safety [3]. High job performance in nurses can not only increase levels of work engagement [4], promote innovation [5], and enhance the meaningfulness of work [6], but also contribute to high levels of job satisfaction and well‐being [7] and reduce turnover intentions and actual turnover [8]. Conversely, poor job performance poses a risk to these benefits, potentially leading to lower quality patient care and increased costs from errors and inefficiencies, and is associated with poor patient outcomes [9]. Maintaining high job performance is therefore essential for the wellbeing, and development of both the nurse and the healthcare organizations [2, 10]. Identification of individual, professional, and environmental factors, especially psycho‐social elements, is essential to improve the job performance of nurses [11].

As well as focusing on the strengths and virtues that enable individuals to flourish, positive psychology emphasizes the importance of beliefs and values in shaping career paths and life achievements [12]. As such a positive psychological belief, belief in free will (BFW) refers to the belief in individual autonomy in decision‐making and action, which is associated with increased positive attitudes, more choices, and higher perceived decision‐making ability [13]. Those with higher BFW are demonstrably enhanced intrinsically motivated, more diligent, proactivity, and tenacious than those with lower BFW [14, 15]. In the workplace, BFW can manifest as a sense of personal responsibility and self‐efficacy, which are key determinants of job satisfaction and performance [16]. Empirical evidence from a range of populations has shown that higher BFW can predict better job performance beyond traditional predictors such as conscientiousness and locus of control [15, 17]. However, research in this area often overlooks the unique challenges of nursing, leaving the impact of BFW on nurses’ motivation and performance largely unexplored, despite the unique demands of the profession, such as emotional labor [18] and high‐stakes decision‐making [19]. Furthermore, the underlying psychological mechanisms linking BFW to job performance remain unknown. Therefore, the present study focused on the association between BFW and job performance in nurses and explored the potential mediation mechanism linking BFW to job performance.

The relationship between BFW and job performance is hypothesized to be mediated by a construct rooted in positive psychology: gratitude. The concept of gratitude involves not only appreciating the help of others but also habitually focusing on and appreciating the positive aspects of life [20]. Previous research has shown that BFW can increase feelings of gratitude by influencing the way people perceive life and the behavior of others [17, 21]. BFW promotes a sense of agency, leading individuals to view positive events as intentional and deserving of appreciation, thereby increasing feelings of gratitude [17]. It has been found that people with stronger BFW reported higher levels of gratitude, and those whose BFW was experimentally weakened felt less grateful for past events and favors [21]. Thus, BFW may be a positive psychological belief that can be shaped to improve an individual’s level of gratitude. In addition, cultivating gratitude in the workplace can promote positive behaviors and outcomes that benefit both employees and organizations [22]. Research has shown that different forms of gratitude—dispositional, collective, and relational—can predict job performance outcomes [23]. Gratitude also fosters collaboration and innovation and improves both task‐ and context‐related performance, especially in healthcare [23, 24]. It plays a critical role in healthcare and is a significant predictor of nurses’ job performance [25]. A scoping review has suggested that gratitude interventions, such as journaling or workshops, can increase new graduate nurses’ well‐being and resilience, reduce turnover, and improve performance [26]. These findings suggest a possible relationship between BFW, gratitude, and job performance in the nursing population, and gratitude may play a mediating role in the relationship between BFW and nurses’ job performance.

In addition to gratitude, BFW can improve job performance through self‐control, which is the ability to manage one’s focus, feelings, actions, and urges to achieve goals in the face of temptations, difficulties, and external demands [27]. BFW can increase an individual’s perceived ability to control their actions and make conscious decisions [28], which in turn increases the ability to resist temptations and impulses and facilitates goal‐directed behavior [29]. Previous studies using the questionnaire measures have shown a positive correlation between BFW and self‐control in various populations, including children [30], college students [31], and the elderly [32]. Evidence from experimental manipulation research has further shown that undermining BFW can reduce self‐control, leading to more impulsive and antisocial behavior [30], which may indicate a causal role of BFW in self‐control. In addition, it is believed that individuals with high levels of self‐control are more likely to be successful in achievement‐related domains, as it has been consistently shown that self‐control has a positive effect on goal pursuit, encompassing the critical processes of goal setting, implementation, monitoring, and resistance to temptation [33]. In particular, exercising self‐control over one’s feelings, thoughts, and behaviors at work is positively linked with achieving organizational goals and successfully completing work tasks [34]. Previous research with geriatric nurses has suggested that self‐control may influence behavioral outcomes such as job stress, burnout, and absenteeism, all of which can directly affect nurses’ job performance [35]. Moreover, it has been suggested that nurses may need to control their emotional responses in order to maintain a professional image or adhere to organizational “display rules,” which are essential to nursing job performance [36]. Altogether, we expected that there may be a mediational relationship between BFW, self‐control, and job performance in nursing populations.

Furthermore, cumulative research has shown that gratitude plays a constructive role in enhancing self‐control. On the one hand, gratitude is associated with an individual’s belief in his or her ability to control events and overcome challenging circumstances, thus fostering a positive mindset for making wiser decisions [37]. Gratitude, as a positive emotional state, may enhance an individual’s ability to delay gratification and exercise self‐control [38]. For example, gratitude has been reported to help individuals shift their focus from immediate desires to long‐term goals and values, promote a sense of satisfaction, and reduce impulsive behavior [20]. Studies have found that nurses who practice gratitude, such as keeping a gratitude journal, show increased levels of self‐control [39]. On the other hand, gratitude helps individuals build the mental and emotional resources needed for self‐control by promoting a positive and expansive mindset [40], which is consistent with the principles of broaden‐and‐build theory [41]. In addition, gratitude can promote empathy and social connectedness and increase one’s sense of responsibility, which further supports self‐control [42]. Considering all of the above, gratitude and self‐control may sequentially mediate the link between BFW and job performance in nurses.

## 2. The Current Study

In this study, we focused on how BFW, gratitude, and self‐control directly or indirectly affect job performance in a group of Chinese nurses and explored the mediation link among these factors. Specifically, we wanted to confirm the relationship between BFW and job performance among nurses, and then uncover the potential psychological mechanisms linking this relationship. Our study aimed to propose and test the following hypotheses: (1) BFW has a positive relationship with nurses’ job performance; (2) gratitude and self‐control independently mediate the relationship between nurses’ BFW and their job performance; (3) gratitude and self‐control sequentially mediate the relationship between nurses’ BFW and their job performance.

## 3. Methods

### 3.1. Participants and Procedure

A cross‐sectional survey was conducted among a convenience sample of nurses from several hospitals in two cities (Chengdu and Kunming) of southwest China between January 2021 and May 2022. The survey used an anonymous online questionnaire that was sent to and distributed by investigators who had received uniform training. These investigators administered the web‐based surveys in their respective hospitals and provided uniform instructions to explain the purpose and significance of the study. Nurses were informed that their participation was anonymous and voluntary, and informed consent was obtained from participants before the study began. Eligibility criteria required that all participants hold a valid nursing qualification certificate from China, have at least 1 year of experience in clinical nursing or clinical nursing management, have basic telephone and computer skills, and have no history of psychosis or drug addiction (assessment by self‐report). Exclusion criteria included nurses in a clinical training program and student nurses. A total of 756 registered nurses who met the inclusion and exclusion criteria participated in our survey. Bogus items with predetermined responses were included in the survey to ensure data quality and identify inattentive participants [43]. Those who answered these items incorrectly, numbering 47, were excluded due to a perceived lack of attention. The final sample therefore included 709 participants.

### 3.2. Measures

#### 3.2.1. Demographic Questionnaire

To assess the demographic and professional details of the participants, the research team designed a comprehensive general data questionnaire covering participant characteristics such as age, nursing experience, gender, marital status, education level, and professional nursing titles.

#### 3.2.2. Free Will Subscale From the Free Will Inventory (FWI)

The FWI was developed to measure individuals’ beliefs about free will and related concepts such as determinism and dualism [44]. The free will subscale of the FWI, which consists of five items assessing the strength of an individual’s BFW, was used in the present study. Each item is rated on a seven‐point Likert scale ranging from 1 (strongly disagree) to 7 (strongly agree), with higher scores indicating stronger BFW. This scale has shown adequate reliability and validity in Chinese populations [45]. In this study, the Cronbach’s alpha coefficient for this scale was 0.78, indicating acceptable internal reliability.

#### 3.2.3. Gratitude Questionnaire‐6 (GQ‐6)

In this study, the Chinese version of the GQ‐6 [46], originally developed by McCullough et al. (2002) [47], was used to measure levels of gratitude. This questionnaire consists of six items, with items 3 and 6 reverse scored to align with the concept of gratitude. Participants were asked to indicate their level of agreement with each statement on a seven‐point Likert scale ranging from 1 (strongly disagree) to 7 (strongly agree). In previous studies, the Chinese version of the GQ‐6 has demonstrated satisfactory reliability and validity in various populations including nurses [48]. In this study, the Cronbach’s alpha coefficient of the GQ‐6 was 0.85, indicating adequate internal reliability.

#### 3.2.4. Brief Self‐Control Scale (BSCS)

In this study, we used the BSCS developed by Tangney and colleagues [49] to assess individual differences in self‐control. The unidimensional questionnaire consists of 13 items, each rated on a five‐point Likert scale ranging from 1 (strongly disagree) to 5 (strongly agree). The BSCS has shown good psychometric properties and has been successfully applied to the Chinese context, providing a reliable and valid instrument for assessing self‐control in Chinese populations including nurses [50]. The Cronbach’s alpha coefficient of the BSCS in our study was 0.80, indicating adequate internal reliability.

#### 3.2.5. Job Performance Scale (JPS)

The JPS is widely recognized in the field of organizational behavior and consists of five key items to assess an individual’s in‐role job performance [51]. The scale uses a seven‐point Likert scale ranging from “strongly disagree” with a score of 1 to “strongly agree” with a score of 7, with the third item scored in reverse. A higher total score indicates a higher perceived level of job performance. The JPS has been successfully applied to the Chinese context, providing a reliable and valid instrument for assessing the ability to perform job responsibilities and perform all duties as required among the Chinese culture [51, 52]. In the present study, the Cronbach’s alpha coefficient for this scale was 0.96, indicating excellent internal reliability.

### 3.3. Data Analysis

The online questionnaire necessitated the completion of every item before it could be submitted, thus precluding any missing data. As a result, the statistical analyses were executed using the unbroken dataset. Given the survey questionnaire‐derived data in this study, we prioritized the assessment of common method bias (CMB) using Harman’s single‐factor test before proceeding with formal data analysis [53]. This involved conducting an exploratory factor analysis with all items of the measures loaded to test for single factor extraction in the unrotated factor solution [54]; the absence of a single dominant factor or its failure to explain more than 40% of the variance indicated that CMB was not a significant concern [53]. For subsequent analysis, we used IBM SPSS 26.0 software enhanced with Hayes’ PROCESS macro to perform mediation analyses [55]. We focused on the relationship between BFW and job performance, with job performance as the dependent variable and BFW as the independent variable. To elucidate the mediating roles of gratitude and self‐control, we applied Model six in the PROCESS macro. This approach allowed us to assess the direct effect of BFW on job performance and the indirect effects mediated by gratitude and self‐control, culminating in an assessment of the total mediation effect. The statistical significance of the mediation effects was determined using bootstrapped 95% confidence intervals (5,000 iterations), with exclusion of zero from the interval indicating a significant mediation effect. Demographic variables (age, sex, education level, and working years) were treated as covariates in the mediation model.

### 3.4. Ethical Considerations and Reporting Guidelines

This study was approved by the Ethics Committee on Biomedical Research, West China Hospital of Sichuan University (approval number: 2021‐1216). All participants were informed of the purpose and use of the study and provided signed informed consent.

To maintain the integrity and transparency of the study, reporting followed the Strengthening the Reporting of Observational Studies in Epidemiology (STROBE) guidelines [56]. For readers seeking a more in‐depth understanding of the STROBE guidelines, further details are available in Supporting Table S1, which provides a comprehensive overview of the criteria and recommendations.

## 4. Results

### 4.1. Demographics and Participant Characteristics

Supporting Table S2 presents the sociodemographic characteristics of the study participants, which included a total of 709 nurses. The majority of participants were female (90.7%), their age ranged from 18 to 55 years, and their nursing experience ranged from 1 to 39 years. More than half of the participants were married (68.1%), had a bachelor’s degree (56.1%), and held a primary professional title (51.6%).

### 4.2. CMB Analysis

We revealed that no single factor or common factor accounted for the majority of the variance in the exploratory factor analysis. Specifically, there were 6 common factors whose values were greater than 1. Notably, the first factor accounted for 18.20% of the variance, which is less than the 40%. These results, while not conclusively ruling out CMB, offer evidence to suggest that it may not represent a substantial threat to the validity of our findings.

### 4.3. Correlation Analysis

Table 1 lists the descriptive statistics and bivariate correlations for the study measures. The results of the bivariate correlation analysis indicated that BFW was positively correlated with job performance (*r* = 0.44, *p* < 0.001), gratitude (*r* = 0.42, *p* < 0.001), and self‐control (*r* = 0.25, *p* < 0.001). In addition, both gratitude (*r* = 0.48, *p* < 0.001) and self‐control (*r* = 0.37, *p* < 0.001) were positively related to job performance. Gratitude and self‐control also showed a positive correlation (*r* = 0.38, *p* < 0.001).

**Table 1:** Descriptive statistics and bivariate correlations of study measures.

Measure	Mean	SD	Skewness	Kurtosis	1	2	3	4
1. Belief in free will	19.39	2.63	−0.09	0.25	—			
2. Gratitude	31.95	5.59	0.06	−0.68	0.42	—		
3. Self‐control	42.82	6.56	0.26	−0.01	0.25	0.38	—	
4. Job performance	29.20	4.21	−0.70	1.06	0.44	0.48	0.37	—

*Note:* All correlation coefficients were statistically significant at the 0.001 level.

Abbreviation: SD, standard deviation.

### 4.4. Mediation Analysis

Table 2 presents the results of mediation analysis, detailing the overall, direct and indirect effects between BFW and job performance. The overall effect of BFW on job performance was significant (*β* = 0.437, *p* < 0.001). When considering the mediating variables, the direct effect of BFW on job performance remained substantial (*β* = 0.265, *p* < 0.001), indicating that BFW has a significant influence on job performance.

**Table 2:** Total, direct, and indirect effects of the mediation model.

Effect	Effect size	Bootstrap SE	Bootstrap 95% CI
**Total effect** (belief in free will ⟶ job performance)	0.437	0.034	[0.371, 0.504]
**Direct effect** (belief in free will ⟶ job performance)	0.265	0.034	[0.198, 0.333]
**Indirect effect**	0.172	0.020	[0.134, 0.212]
Belief in free will ⟶ gratitude ⟶ job performance	0.126	0.018	[0.091, 0.163]
Belief in free will ⟶ self‐control ⟶ job performance	0.021	0.009	[0.004, 0.041]
Belief in free will ⟶ gratitude ⟶ Self‐control ⟶ job performance	0.025	0.007	[0.013, 0.041]

*Note:* Standardized coefficients were used to estimate the effect sizes.

Abbreviations: CI, confidence interval; SE, standard error.

**Figure 1:**
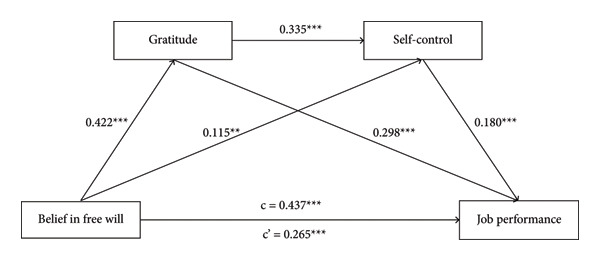
The mediating effects on belief in free will and job performance. Demographic variables (age, sex, education level, and working years) were treated as covariates in the mediation model. Standardized regression coefficients were displayed in the path diagram; c, total effect; c’ direct effect; ∗∗∗*p* < 0.001, ∗∗*p* < 0.01.

As shown in Table 2 and Figure 1, the mediating effects of gratitude and self‐control on the relationship between BFW and job performance were significant. First, gratitude was found to be a significant mediator in the relationship between BFW and job performance (effect size = 0.126, *p* < 0.05). This indirect effect represents a medium effect size, based on the guidelines for what constitutes large (*β* > 0.30), medium (0.10 ≤ *β* ≤ 0.30), and small (*β* < 0.10) effect sizes [57]. In addition, self‐control also emerged as an independent mediator (effect size = 0.021, *p* < 0.05). This indirect effect represents a small effect size [57], indicating a modest contribution of self‐control in mediating the link between BFW and job performance. Furthermore, the serial mediation analysis revealed that the effect of BFW on job performance was further strengthened through the sequential pathway involving gratitude and self‐control (effect size = 0.025, *p* < 0.05). This indirect effect also represents a small effect size [57], suggesting that the sequential mediation of gratitude and self‐control contributes a small, but significant, portion to the BFW‐job performance link. These results suggest that BFW not only directly influences job performance, but also indirectly influences it by promoting a sense of gratitude and enhancing self‐control, which together contribute to improved job performance.

## 5. Discussion

This study examined the link between BFW and job performance in nurses, highlighting the direct and indirect effects of BFW on job performance. We first revealed that there was a reliable positive association between nurse’s BFW and job performance. Then we found that gratitude and self‐control served an independent and sequential mediator in the BFW‐job performance association. Overall, our findings confirm the positive relationship between BFW and job performance in nurses and provide novel evidence for the underlying psychological mechanism linking BFW to job performance.

First, our research revealed a moderate to large positive correlation between BFW and job performance in nurses, consistent with prior research in other occupational groups [15]. This relationship is likely explained by several key psychological mechanisms linked to BFW. Specifically, BFW is crucial for setting and pursuing meaningful goals [58] and promoting persistence [59]. The increased goal‐directed motivation associated with BFW can translate to higher performance [60]. BFW facilitates proactive decision‐making and effective responses to changing situations [61, 62], enabling nurses to cope with workplace challenges [63]. By reducing indecisiveness and promoting decisive action [64], BFW can improve the quality and efficiency of clinical judgments. Furthermore, BFW serves as adaptive coping resources that promote positive attitudes and desirable behaviors [45, 65], which aids in stress management and adaptation within the demanding healthcare environment. The link between BFW and biological adaptation suggests a foundation for socially desirable behaviors and successful interpersonal interactions [66, 67]. Besides, BFW promotes agency and responsibility [44], potentially increasing intrinsic motivation and professional commitment [15]. These benefits, in turn, improve job performance and patient care quality [68]. Therefore, BFW is linked with better nursing performance by influencing goal pursuit, clinical decision‐making, adaptive behaviors, and intrinsic motivation. These combined effects may promote work effectiveness and professional advancement.

Second, our research found that BFW indirectly influences nurses’ job performance through gratitude. This suggests that fostering BFW may lead to positive emotions of gratitude, which in turn contribute to favorable behaviors and organizational outcomes [48]. Our findings are generally consistent with theoretical frameworks that posit an association between BFW and an increased sense of personal responsibility and appreciation for positive events [16]. Nurses with higher BFW are more likely to perceive positive events and acts of kindness as intentional choices, fostering feelings of gratitude and reciprocity [69, 70]. This can enhance prosocial motivation and cooperation in professional settings [71, 72], leading to positive team dynamics and improved patient care [73]. Gratitude also helps individuals develop psychological resources and effective coping strategies, which result in positive workplace outcomes [48]. As a positive emotion, gratitude expands thought‐action repertoires, encouraging creative problem‐solving [74]. Nurses with higher gratitude demonstrate greater innovative capacity [75], leading to improved job performance [76]. Practically, encouraging gratitude through workplace interventions like gratitude journals or team‐based appreciation exercises could improve collaboration and innovation in nursing teams [26], ultimately enhancing job performance and patient care. In sum, the current findings expand our understanding of the relationship between BFW, gratitude, and job performance and highlight the importance of gratitude in improving job performance.

Third, our study showed that BFW helps nurses improve their job performance by improving self‐control. This aligns with theories that conceptualize BFW as a precursor to intentional action and self‐regulation [16, 77]. Individuals with a strong belief in their agency are more likely to feel responsible for their actions and better control their behaviors, ultimately improving job performance [78]. The mediating role of self‐control is especially important in the challenging nursing environment. Nurses must effectively manage stress, regulate emotions, and adhere to protocols under pressure [79]. Self‐control enables nurses to make rational decisions, prioritize tasks, and remain resilient in the face of adversity [49]. Nurses with a strong BFW may perceive their ability to control their actions as a reflection of their agency [77], empowering them to perform their work consistently and effectively [80, 81]. Self‐control is also associated with increased work engagement, lower burnout, and adherence to professional standards [82, 83]. The ability to regulate emotions and resist distractions may contribute to improved focus and concentration, allowing nurses to provide optimal patient care. In practice, strategies to enhance self‐control, such as mindfulness training or time management workshops [84], could help nurses better manage the demands of their jobs, leading to improved performance and reduced stress. Therefore, our findings suggest that self‐control may serve as a critical link between BFW and nurses’ job performance.

Finally, this study found that gratitude and self‐control sequentially mediate the relation between BFW and nurses’ job performance. This serial mediation highlights a cascading effect where BFW initiates a positive psychological chain reaction that promotes intrinsic motivation and adaptive behavior [16]. Gratitude, arising from appreciating others or the profession [68, 70], appears to act as a foundation for self‐control. Nurses who experience gratitude are more attuned to the positive aspects of their work, enhancing intrinsic motivation and their ability to regulate their responses [49, 85]. This allows for more effective decision‐making, task prioritization, and emotional and physical management, all critical in nursing [86]. Nursing requires commitment, emotional management, and behavioral control, so nurses with high gratitude may be better equipped to activate self‐control mechanisms. This suggests that interventions targeting gratitude could indirectly boost self‐control, leading to a synergistic improvement in job performance. For example, creating a supportive work environment that encourages recognition and appreciation could foster gratitude and subsequently enhance self‐control [87]. The serial nature of the mediation suggests that gratitude potentiates the effects of self‐control, enhancing the positive influence of BFW. Gratitude may strengthen nurses’ motivation to perform well, while self‐control provides the means to achieve the desired behaviors and decisions.

This study has some limitations. First, the cross‐sectional nature of the research means that we cannot make causal inferences about the relationships between the variables. The conclusions drawn are correlational, and we need a longitudinal approach to establish causality. Second, all data were collected using self‐report measures. While Harman’s single‐factor test did not point to substantial CMB, the reliance on self‐report measures might have introduced bias [53]. In the future, researchers could use more objective data collection methods, such as ecological momentary assessment or the experience sampling method [88], to reduce the impact of reporting bias. Third, the mediation model in this study centered on gratitude and self‐control as key mediators connecting BFW to job performance. Nevertheless, other self‐regulatory traits, such as self‐efficacy, conscientiousness, and resilience, may also act as mediators. Given their conceptual overlap with gratitude and self‐control, these traits could independently contribute to job performance. Examining these alternative mediators has the potential to considerably broaden the research’s theoretical scope. Finally, our sample’s confinement to nurses in two Chinese cities, coupled with a gender imbalance (predominantly female) and a lack of educational diversity (most holding bachelor’s degrees), may hinder the generalizability of our findings to other populations and healthcare settings. Consequently, careful consideration is warranted when extrapolating these results to different countries, healthcare systems, or nursing specialties. To better understand the impact of agency beliefs on professional resilience and performance in healthcare, studies employing comparative methodologies and multiple data sources are recommended. Moreover, the study’s execution during the COVID‐19 pandemic introduced a unique context that could limit the applicability of our findings. The increased stress, workload, and infection risks experienced by nurses during this period may have disproportionately affected the relationships between BFW, gratitude, self‐control, and job performance [89.] Future research should examine these relationships in varied contexts to assess the pandemic’s influence on our results.

## 6. Implications for Nursing Management

Our findings have significant implications for nursing administration, contributing to a better understanding of the psychosocial factors influencing nurses’ job performance. Recognizing the impact of psychosocial factors like BFW, gratitude, and self‐control allows nurse administrators to develop strategies to boost staff effectiveness and patient care. By cultivating a positive psychological climate, nurse leaders can encourage nurses to achieve higher levels of performance. This could involve promoting a culture of autonomy and decision‐making (to strengthen BFW), implementing gratitude‐based interventions, and providing resources to improve self‐control. It is also recommended that nursing education programs integrate modules on positive psychology to foster continuous growth in job performance.

## 7. Conclusion

To conclude, this study investigated the connection between BFW and job performance among nurses, as well as the mediating mechanisms of gratitude and self‐control. The findings indicated a positive relationship between nurses’ BFW and job performance. Moreover, gratitude and self‐control were found to independently and serially mediate this relationship. These results expand our knowledge regarding the link between BFW and job performance, emphasizing the value of cultivating gratitude and self‐control to enhance job performance in nurses.

## Ethics Statement

The local Ethics Committee on Biomedical research, West China Hospital of Sichuan University approved the present study (approval number: 2021‐1216). All participants read and signed an online informed consent form before the research.

## Conflicts of Interest

The authors declare no conflicts of interest.

## Author Contributions

Linli Zhuang: data collection, investigation, formal analysis, writing–original draft. Xueling Suo: conceptualization, methodology, software, validation. Song Wang: conceptualization, project administration, resources, supervision, writing–review and editing. Linli Zhuang and Xueling Suo are the co‐first authors of this paper.

## Funding

This study was supported by the Key Research and Development Program of Sichuan Province (Grant No. 2023YFS0084).

## Supporting Information

Table S1. STROBE Statement—Checklist of items that should be included in reports of cross‐sectional studies.

Table S2. Sociodemographic characteristics of the participants.

## Supporting information


**Supporting Information** Additional supporting information can be found online in the Supporting Information section.

## Data Availability

The data that support the findings of this study are available from the corresponding author upon reasonable request.
